# Effect of integrated yogic practices on positive and negative emotions in healthy adults

**DOI:** 10.4103/0973-6131.78174

**Published:** 2011

**Authors:** Lakshmi Narasimhan, R Nagarathna, HR Nagendra

**Affiliations:** Division of Life Sciences and Yoga, Swami Vivekananda Yoga Aunsandhana Samsthana (SVYASA), Jigani, Bangalore, Karnataka, India

**Keywords:** Negative affect, one week, positive affect, yoga

## Abstract

**Background:**

Studies on affective wellbeing have shown the beneficial role of positive emotions on cognitive processing and the harmful role of negative emotions on coping, stress and health status. Studies have shown that yoga practices reduce anxiety and depression and improve wellbeing.

**Objective:**

The aims of the study were to, (i) examine the safety and feasibility of conducting a weeklong free yoga camp, and (ii) assess its impact on the negative and positive affect in normal healthy volunteers.

**Materials and Methods:**

In this open-arm study450 participants were taught integrated yoga module. It included asanas, pranayama, relaxation, notional correction and devotional sessions. Assessment was carried out on the first and last day of the camp, using a modified version of Positive Affect Negative Affect Scale (PANAS). It has ten questions each to measure positive (PA) and negative affect (NA). Nine questions have been added which are referred as other positive affect (OPA) and other negative affect (ONA) domains.

**Results:**

Three hundred and twelve sets of pre–post data were analyzed. There was an increase in PA of PANAS by 13% (*P*<0.001, Wilcoxon’s signed rank test) and OPA by 17% (*P*<0.001). The NA reduced by 47% (*P*<0.001) and ONA by 48% (*P*<0.001).

**Conclusion:**

It is feasible and safe to conduct a weeklong yoga camp in an urban setting, and integrated yoga practices can reduce the negative affect and increase the positive affect within one week.

## INTRODUCTION

In present-day lifestyle, although modern technology has helped to protect us from physical damages like injuries, infections, accidents, we are faced with many emotionally demanding situations in all fields of life, such as high-level competition, unemployment, unending and ever-mounting targets, high expectations at the workplace, adjustments in families, dealing with difficult personalities, etc. causing heightened anxiety and stress.[1] Stress is not viewed as a singular event, but as a transaction between an individual and the environment that makes demand on all available coping resources of the body-mind complex. This involves cognitive appraisal and coping processes. When these resources are taxed and the responses exceed the coping abilities it can result in distressful negative emotions.[2] These precipitate aggressive behaviors such as anger, fear, distress, irritability etc. Stress and coping are closely related to affect or emotions because they are affected by cognitive appraisal.[3] Thus the heightened stress responses that result in negative affect and distress, are reflections of an inability to cope with demanding situations.[4]

An emotion is defined as a mental and physiological state associated with a wide variety of feelings, thoughts, and behaviors. It is a prime determinant of the sense of subjective wellbeing and appears to play a central role in many human activities.[5] Watson *et al*., measured these emotions under two major categories namely positive and negative affect. Pleasant emotions of different intensities may be grouped as ‘positive affectivity’ (PA) and unpleasant emotions under ‘negative affectivity’ (NA).[6]

### Negative affect

Negative affect (NA) is the dimension with aversive mood states and subjective distress. Itlowers self-esteem, depresses the quality of relationships with others, and leads to anxiety and depression that narrows the attention.[7] NA as fear facilitates withdrawal behavior in situations that threaten survival[7] which depends on the capacity to cope with different situations.[8] A state of calmness and contentment are characterized by low NA.[6]

### Positive affect

Those with greater tendencies to cope through humor reported greater positive mood, and have shown increased levels of salivary immunoglobulin A (S-IgA), a vital immune system protein.[9] Positive emotions, especially hope, may uniquely contribute to the health benefits accrued by dispositional optimists.[4]

### Remedial measures

Coping strategies related to the occurrence and maintenance of positive emotions (e.g., positive reappraisal, problem-focused coping, and infusing ordinary events with positive meaning) help buffer against stress and depressed mood.[10] These strategies help individuals emerge from crises with new coping skills through closer relationships, and a richer appreciation for life, all of which predict an increase in psychological wellbeing.[11] Studies have shown the benefits of positive affect in prevention and rehabilitation of stress-related diseases like hypertension,[12] gastrointestinal disorders,[13] coronary heart disease[14] and diabetes parameters. Higher positive affect predicted lower levels of glycosylated hemoglobin in normal people, indicating the beneficial effect of positive affect on diabetic parameters.[15]

### Yoga

Yoga is one of the popular practices that has the potential to promote positive affect. According to a recent survey in 2008, conducted by the National Centre for Complementary and Alternative Medicine, 38% percent of adults and 12% of children in USA use complementary and alternative therapies and yoga is one amongst the top five of these.[16] Yoga, developed thousands of years ago, is now recognized as a form of mind-body medicine. It addresses the full scope of a person’s life including physical, mental, emotional and spiritual aspects of the individual in disease and health.

Reduction in negative affect with yoga has been shown to improve depression and anxiety amongst distressed women.[17] Also, yoga has been used effectively in reducing pain and increasing flexibility in chronic low back pain,[18] to combat stress in cancer patients,[19] to increase lung functions and reduce usage of bronchodilator in asthmatics[20] and for rehabilitation of patients with post-stroke hemiparesis.[21]

The benefits of reduction in negative emotions can improve the quality of life in healthy people with increased immunity,[9] better pulmonary functions[22] and increased lifespan.[23]

#### Yoga and physical health

In young trainees yoga improved joint flexibility,[24] respiratory endurance and muscle strength[25] and also dexterity in students.[26] Reduction in body fat, improved shoulder flexibility in elderly females,[27] improvement in immunological tolerance,[28] favorable changes in neuro-endocrine functions including melatonin and cortisol secretions[29–31], lower perceived exertion after exhaustive exercise[32] are the other documented physical health benefits of yoga.

#### Yoga for positive mental health

Improved cognitive functions have been reported in children and adults after practicing integrated yoga. Increased visual perception,[33] better learning skills[34] and increase in spatial and verbal memory[35] have been demonstrated within 10 to 30 days of yoga practices.

#### Yoga for positive emotional health

Studies that have assessed the emotional states by ‘Profile of Mood States’ after yoga have reported significant improvements in negative emotions including tension-anxiety, depression-dejection, anger-hostility, fatigue-inertia, and confusion-bewilderment.[17,36] A short-term Iyengar yoga (10 h) has shown improvement in self-reported acute mood states of depression trait anxiety, negative mood and fatigue in young adults.[37] A study that compared African dance and Hatha yoga showed reduced perceived stress and negative affect with both these practices but reduced cortisol levels in the hatha yoga group.[30] The utility of yoga in improving mood and the differential effects may be related to its influence on physiological states of arousal[30] through establishing stable autonomic balance.[38]

Thus, ameliorating negative affectivity and increasing positive affectivity is one of the main concerns in stress management. In the present study, we examined the positive and negative affect outcomes after a short-term intervention of integrated yoga in normal adults who volunteered to attend a yoga camp.

## MATERIALS AND METHODS

### Participants

Nine hundred and forty participants in the age range of 13-78 years volunteered to attend a weeklong non-residential free yoga camp in response to public advertisements in the city of Patna, India. Those who had health problems as per their statement in their registration were excluded. Thus, 450 participants were included in the positive health program designed for normal healthy participants of both sexes. The inclusion criteria were participants from both sexes between the age group of 15-78 years and those who could read and write Hindi or English language. Signed informed consent was obtained from all participants before the camp started.

### Design

This was an open-armed observational study in a naturalistic setting to evaluate the changes in positive and negative emotions after yoga intervention in participants who attended the weeklong free yoga camp at Patna, India. The camp was advertised through banners, local newspapers and local TV channels. The camp was conducted from 1^st^ to 7^th^ November 2006. The classes were conducted from 6 to 8 am and 6 to 8 pm, in the centre of the city to suit the convenience of people. After checking the registration forms for inclusion and exclusion criteria, the participants were divided into different groups based on their age and sex. All participants recruited for the study were assessed on the first and last day of the camp using modified Positive Affect Negative Affect Questionnaire (PANAS). The questionnaire was printed in both Hindi and English. After reading out the instructions on the public address system, they were asked to fill up the sheets carefully. The group instructors were monitoring the entire procedure in their groups. The instructors took care not give any interpretation of the questions.

### Intervention

The instructions with demonstrations for the practices were given from a common raised platform on the public address system by the senior yoga faculty of VYASA. Each group was supervised by two to three assistant instructors for corrections of the practices. These instructors developed a good rapport with their group and enquired about any negative affect about practices.

The classes began at 6 am with a prayer followed by loosening exercises (*Çithilékarëa vyäyäma*) and breathing exercises for 20 min. Then, there was a talk followed by the practice of *praëäyäma* for 40 min with 10 min for questions and answers. This was followed by *Suryanamaskära* and Yoga postures that were introduced systematically (40 min). This session of physical practices ended with guided deep relaxation technique in supine position for 10 min. The sessions ended with a closing prayer. The evening sessions (6-8 pm) comprised of devotional sessions for emotion culture, followed by lectures and answers to written questions based on the previous day’s lecture.

### Assessment

Assessment was done using the PANAS questionnaire developed by Watson *et al*.[6] The PANAS is a 20-item questionnaire designed to measure positive and negative affect. It has ten questions each to measure positive and negative emotions, referred to as positive affect (PA) and negative affect (NA). The internal reliability (Cronbach’s coefficient alpha) is 0.86 to 0.96 for positive affect and 0.84 to 0.87 for negative affect of the PANAS.[6] To tap other aspects of emotions relevant to the Indian population, we added nine (four positive and five negative) questions for this study which are referred as Other Positive Affect (OPA) and Other Negative Affect (ONA). The PANAS and OPANAS domain scores were analyzed and interpreted separately since the questions that were added had not been tested earlier for validity and reliability.

### Data extraction

The participants rated all questions on a 5-point scale of 0-4.

(0=not at all, 1=a little, 2=moderately, 3=quite a bit, 4=extremely) reflecting the extent to which they experienced the emotion during the past one week. All 29 questions were intermixed in the questionnaire. They were carefully isolated for obtaining the individual scores for the four domains i.e. PA, NA, OPA and ONA. Incomplete answer sheets were discarded.

### Data analysis

Statistical analysis was done using ‘SPSS, 10’ software. Normality was checked using Kolmogorov Smirnov test. As the dataset was not normally distributed, Wilcoxon’s test was used to compare the pre-and post-values.

## RESULTS

Of the 450 participants who satisfied the selection criteria for promoting positive health, 355 were available on the morning of the last day of the camp to answer the questionnaire and 312 suitable sets of pre and post answer sheets were available for final analysis.

Reasons for dropout were incomplete answer sheets and inability to attend all classes due to various reasons. One of the reasons for poor attendance appeared to be lack of commitment as it was a free camp.

Table 1 shows the demographic data of the participants. Out of 312 participants, 78% were below 25 years and 22% were above 25 years of age. 0nly 8% of the participants were females.

**Table 1:** Demographic Data

Variable	Number
Total	312
Males	287
Females	25
Age in years	
15-20	200
21-25	55
>25	57
Age range (in years)	15-78
Age mean±SD (in years)	24.92±13.85
Occupation	
Students	249
Working men	38
Working women	04
Housewives	21
Retired	09
Education	
School	252
Graduates	55
Postgraduates	05

Table 2 shows the changes in PANAS after yoga. There was a significant improvement in positive affect after yoga at a *P*<0.001, showing 13% and 17% changes in PA and OPA respectively. The NA decreased after yoga at a *P*<0.001, with 47% and 48% reduction in NA and ONA respectively.

**Table 2:** Results of integrated yoga practices in normal volunteers

Variables	Mean±SD		95% CI of mean pre		95% CI of mean post		Pre-post Wilcoxon’s *P* value	% changes
	Pre-yoga	Post-yoga	Lower bound	Upper bound	Lower bound	Upper bound		
PANAS positive	23.47±7.04	26.54±6.37	22.68	24.25	25.83	27.25	<0.001	+ 13
Other positive	9.33±3.42	11.28±2.98	8.95	9.71	10.95	11.61	<0.001	+ 17
PANAS negative	12.51±8.25	6.62±6.39	11.59	13.43	5.91	7.33	<0.001	−47
Other negative	7.36±4.99	3.86±3.75	6.80	7.91	3.44	4.28	<0.001	−48

Table 3 shows the changes in individual items of positive affect domains (PA and OPA). There was an increase ranging from 3-21% in the individual items of PA with a negative change -3% in the question ‘excitement’. There was 17-28% increase in the OPA scores. Question number 15 (‘content’) indicating the degree of contentment showed the highest degree of improvement (28%.)

**Table 3:** Changes in individual items of positive affect after yoga practices

Question no	Panas positive affect	
	Descriptor	% Change (increase)
Positive affect		
2	Attentive	16
3	Interested	12
7	Excited	decrease - 3
10	Strong	21.0
11	Enthusiastic	03.0
17	Determined	12.0
18	Proud	16.0
22	Inspired	17.0
25	Active	19.7
29	Alert	13.5
Other positive affect		
1	Happy	18.0
8	Pleased	19.7
15	Content	28.0
26	Glad	17.0

Table 4 shows the changes in the individual items of the negative affect. It is noteworthy that the degree of changes in the negative affect is better, in the range of 38-55%, than the increase in the items on positive affect. The NA descriptor ‘Irritability’ showed the maximum reduction of 55%.

**Table 4:** Changes in individual items of negative affect after yoga practices

Question no.	Panas negative affect	
	Descriptor	% Change (decrease)
Negative affect		
4	Afraid	44.00
6	Distressed	49.00
9	Upset	38.00
12	Jittery	43.00
14	Guilty	47.90
16	Nervous	46.70
20	Scared	43.02
21	Hostile	45.00
24	Ashamed	49.70
28	Irritable	54.90
Other negative affect		
5	Disappointed	47.00
13	Sad	49.33
19	Unhappy	46.66
23	Troubled	46.00
27	Miserable	47.81

## DISCUSSION

### Summary

This open-armed observational study on 312 participants of a weeklong free yoga camp for promotion of positive health through integrated yoga practices showed significant reduction in negative affect and increase in positive affect scores on modified version of PANAS questionnaire.

#### Probable mechanisms and correlation with previous findings

The descriptive of negative emotions, ‘guilty’ and ‘ashamed’ showed 48% and 45% reduction respectively. Since the maximum number of participants in this study group were below the age of 25 years (78%), it points to the beneficial effect of the integrated yoga module in unwinding the guilt feeling in young students within a short period that may be considered an important contribution of this study. Yoga techniques that are meant to develop better mastery over the modifications of the mind (*yogah chitta vrtti nirodhah* as defined by the sage Patanjali)[39] through introspective awareness to calm down the mind (*manah prashamana upayah* as defined by sage *Vasishtha*),[40] may have increased their level of confidence to make a resolve to change themselves and overcome their guilt, shame and the related complexes. Similar changes have been reported in a study after Vipassana meditation in Tihar Jail. The inmates of the jail showed reduced hostility, anxiety and depression with improved sense of wellbeing and hope for the future in those with or without psychiatric problems.[41] Reduction in aggressive behavior has been demonstrated in normal young volunteers after 12 weeks of integrated yoga program similar to the practices used in this study.[42]

Emotions such as ‘Jittery, Nervous, Afraid and Scared’, may all be looked upon as different degrees of performance anxiety that is a very common stress response to academic and psychosocially demanding situations in a progressive society. Many studies have shown the stress-reducing effect of yoga[17,19,30] which support the observation of the present study.

The relaxation response after yoga may offer the ability to face the situations in a relaxed state of mind and perform with utter ease and effortlessness. This is described as one of the quoted definitions of yoga, ‘*yogah karmasu kaushalam*’ by *Sri Krsna in the Bhagavadgita*,[43] which means ’yoga is a special skill of action in relaxation’. This was observed with yoga practices in musicians with the relative reduction in performance anxiety, musculoskeletal conditions, mood and flow experience.[44] Yoga practices prior to exams in medical students showed improved concentration, improved efficiency, increased attentiveness, and significant reduction in number of failures.[45]

‘Disappointed, upset, irritable, hostile’ are different facets of anger resulting from unsatisfied desires or the inability to cope. All this is described in the Bhagavadgita as violent speed of mind resulting in anxiety or depression. These have shown reduction in this study. Benefits of yoga practices for rapid stress reduction and anxiolysis among distressed women,[17] betterment of mood in psychiatric inpatients,[36] and reduction in symptoms of depression[37] are reported.

The perception of vigor ‘Strong’ and ‘active’ (q.10, 25) have increased by 21% and 20% respectively. The feeling of wellness was contributed by asanas and loosening exercises which increases spinal flexibility,[24] dexterity[26] and stamina.[25]

The integrated yoga program taught in this camp included lectures and practice of bhakti yoga (devotional sessions) that are meant for direct handling of emotions by nurturing the positive emotions of pure love and surrender to the divine as tools for stress reduction and positive health.[46] Similar thinking is expressed by a study, which said that spirituality (faith, selfless service and pure love) promotes a healthier coping style.[47] An increase in positive affect ‘contentment’ by 28% and the reduction in the positive affect ‘excitement’ by (-3%) reflects the calming effect of yoga rather than a stimulated happy state of excitement.

## CONCLUSION

Integrated yoga can be taught to normal participants without any harmful effects and it may reduce the negative affect and increase the positive affect within a week.

### Limitations of the study

- Since this was an open-armed observational study in a free camp, the conclusions from this are only pointers rather than evidence-based conclusions.
- The questions of OPA and ONA are not validated.
- High dropout due to the nature of the free camp where it was not possible to control the attendance.

### Strength of this study

This study provides evidence for the feasibility of conducting camps ‘yoga for promotion of positive health’ in a city where people can be taught yoga practices during the working days. The camp attracted a good number of students, which is a welcome sign that yoga is acceptable to healthy youngsters. The observation that there could be significant changes after a weeklong program supports the utility of such free camps which have become very popular in India.

### Suggestions for future work

Randomized controlled studies are necessary to confirm these results. Future studies may also incorporate other psychological and objective measures of mood and emotions to understand the mechanisms.
